# Margret M. and Paul B. Baltes Award Lecture: Optimism and Health: Resource or Delusion?

**DOI:** 10.1093/geroni/igab046.1567

**Published:** 2021-12-17

**Authors:** Eileen Crimmins

## Abstract

The lecture will be given by the 2020 Baltes Award recipient, William Chopik, PhD, of Michigan State University. The recipient of the 2021 Baltes Award is Laura B. Zahodne, PhD, of the University of Michigan. The Margret M. and Paul B. Baltes Foundation Award in Behavioral and Social Gerontology recognizes outstanding early-career contributions in behavioral and social gerontology. The award is generously funded by the Margret M. and Paul B. Baltes Foundation.

